# Diagnostic utility of C-reactive Protein combined with brain natriuretic peptide in acute pulmonary edema: a cross sectional study

**DOI:** 10.1186/1465-9921-12-83

**Published:** 2011-06-22

**Authors:** Kosaku Komiya, Hiroshi Ishii, Shinji Teramoto, Osamu Takahashi, Nobuoki Eshima, Ou Yamaguchi, Noriyuki Ebi, Junji Murakami, Hidehiko Yamamoto, Jun-ichi Kadota

**Affiliations:** 1 Department of Internal Medicine 2, Oita University Faculty of Medicine, 1-1 Idaigaoka, Yufu (879-5593), Japan; 2 Department of Respiratory Medicine, Graduate School of Comprehensive Human Sciences, University of Tsukuba, Hitachinaka Education and Research Center, 20-1 Ishikawa, Hitachinaka (317-0077), Japan; 3 Center for Clinical Epidemiology, St. Luke's Life Science Institute, 10-1 Akashi-machi, Chuo (104-0044), Japan; 4 Department of Biostatistics, Oita University Faculty of Medicine, 1-1 Idaigaoka, Yufu (879-5593), Japan; 5 Departments of Respiratory Medicine, Aso Iizuka Hospital, 3-83 Yoshio-machi, Iizuka (820-0018), Japan; 6 Department of Radiology, Aso Iizuka Hospital, 3-83 Yoshio-machi, Iizuka (820-0018), Japan

## Abstract

Introduction Discriminating acute lung injury (ALI) or acute respiratory distress syndrome (ARDS) from cardiogenic pulmonary edema (CPE) using the plasma level of brain natriuretic peptide (BNP) alone remains controversial. The aim of this study was to determine the diagnostic utility of combination measurements of BNP and C-reactive protein (CRP) in critically ill patients with pulmonary edema.

**Methods:**

This was a cross-sectional study. BNP and CRP data from 147 patients who presented to the emergency department due to acute respiratory failure with bilateral pulmonary infiltrates were analyzed.

**Results:**

There were 53 patients with ALI/ARDS, 71 with CPE, and 23 with mixed edema. Median BNP and CRP levels were 202 (interquartile range 95-439) pg/mL and 119 (62-165) mg/L in ALI/ARDS, and 691 (416-1,194) pg/mL (p < 0.001) and 8 (2-42) mg/L (p < 0.001) in CPE. BNP or CRP alone offered good discriminatory performance (C-statistics 0.831 and 0.887), but the combination offered greater one [C-statistics 0.931 (p < 0.001 versus BNP) (p = 0.030 versus CRP)]. In multiple logistic-regression, BNP and CRP were independent predictors for the diagnosis after adjusting for other variables.

**Conclusions:**

Measurement of CRP is useful as well as that of BNP for distinguishing ALI/ARDS from CPE. Furthermore, a combination of BNP and CRP can provide higher accuracy for the diagnosis.

## Introduction

Acute hypoxic respiratory failure due to pulmonary edema is a common reason for visiting the emergency department. The distinction between cardiogenic pulmonary edema (CPE) and acute lung injury (ALI) or acute respiratory distress syndrome (ARDS) is clinically important because the management and the prognosis are different [1]. Hence, possible biomarkers for the differential diagnosis have been investigated using various strategies [2-5]. The most widely used clinical definition of ALI/ARDS is based on the acute onset of respiratory failure, bilateral infiltration on chest radiography, a pulmonary capillary wedge pressure (PCWP) < 18 mmHg, or absence of clinical evidence of elevated left atrial pressure [2]. However, clinical estimation of PCWP is notoriously inaccurate [6], relatively invasive and costly [7], has no clear evidence of benefits [8,9], and may result in potentially adverse clinical outcomes [10]. Moreover, ALI/ARDS with concomitant heart failure complicates the differential diagnosis [11,12]. If the respiratory status rapidly improves after the administration of diuretics alone, CPE should be considered, whereas patients who have poor responses to sufficient diuresis should be considered to have non-cardiogenic pulmonary edema. In patients with CPE that was triggered by an airway infection, the respiratory status might not completely improve in response to diuretic treatment, if airway an infection itself influences this status to some degree. Therefore, these patients who cannot undergo invasive examinations, such as bronchoscopy because of poor respiratory conditions, tend to only be diagnosed after the start of treatment [13-16]. However, making an accurate initial diagnosis is still important in the emergency department, and the alternative tools for use in maing a differential diagnosis have been explored using various strategies [17], including the measurement of the alveolar protein concentration [5], as well as the use of chest radiographs [13,14] and echocardiography [18].

Brain natriuretic peptide (BNP) is released from the cardiac ventricles in response to increased cardiac wall tension. Rapid measurement of BNP has been shown to be a sensitive marker of dyspnea due to cardiac causes in the emergency room and intensive care unit settings [19-21]. In critically ill patients with hypoxic respiratory failure due to CPE versus ALI/ARDS, the diagnostic utility of BNP has been extensively investigated [15,16,22], however, the accuracy of discriminating these two disorders by the BNP level alone is still a matter of debate. Several investigators have reported that BNP levels do not accurately discriminate CPE from sepsis [23-25]. Severe sepsis, known as one cause of ALI/ARDS, can increase BNP levels in spite of a normal cardiac function [24]. Therefore, false-positive findings of plasma BNP levels may be found in sepsis-associated ALI/ARDS patients.

On the other hand, C-reactive protein (CRP) is an acute phase protein produced primarily from the liver and is stimulated by the release of cytokines, such as interleukin-6 [26]. CRP is a marker of systematic inflammation that is elevated by a wide variety of diseases [27,28], and is widely-used at numerous emergency departments. The severe inflammatory process of the lung in ALI/ARDS patients occurs in response to various etiologies, including pulmonary or extrapulmonary injury [29]. Although there have been a few reported studies regarding the CRP levels [27,30] in critically ill patients with ALI/ARDS, the differential diagnostic value of CRP for these conditions has not been determined. Furthermore, the utility of using a combination of BNP and CRP has not been examined for the differential diagnosis of ALI/ARDS and CPE.

We therefore assessed the diagnostic utility of measuring the plasma BNP levels combined with CRP levels in patients with hypoxic acute respiratory failure due to CPE versus ALI/ARDS.

## Materials and methods

### Setting and patients

This was a single-center cross-sectional study. The protocol was approved by the institutional review boards of the Aso Iizuka Hospital, and informed consent for participation was obtained from each patient or a surrogate decision maker. The hospital is a large teaching hospital with 1,600 inpatient beds. The emergency physicians routinely measured BNP and CRP levels in the plasma of the emergency outpatients, who met the following criteria: presentation with acute respiratory failure with a PaO_2_/fraction of inspired oxygen (FiO_2_) ≤ 300 [2], bilateral pulmonary infiltrates on chest radiography, and age ≥18 years, between May 2004 and March 2010 at the emergency department. The patients who had the following diagnoses or disease conditions on admission were excluded from this study: previously-detected interstitial pneumonia, severe bronchial asthma (stage III or more severe disease based on the criteria of the Global Initiative for Asthma [31]) or chronic obstructive pulmonary disease (stage III or more severe based on the criteria of the Global Initiative for Chronic Obstructive Lung Disease [32]), distinct acute coronary syndrome, renal failure requiring dialysis, cardiac surgery within 2 months, a preexisting decrease of the left ventricular ejection fraction (LVEF: < 30%), malignancies such as lung cancer and lymphoma, intracranial hemorrhage, or cardio-pulmonary arrest in the emergency room.

We designed the study to have a 90% power (β-level = 0.01) with an α-level of 0.05 to show that the AUC of 0.75 for either the CRP or BNP test is significant from the null hypothesis value of 0.5. A total of 106 subjects (53 subjects in each group) are required [33].

The baseline characteristics recorded at the time of enrollment included the following: patient demographics, past medical history including affectors for CRP producibility (e.g. chronic hepatic failure and receiving corticosteroids), blood pressure, heart rate, body temperature, S3 gallop, PaO_2_/FiO_2_, white blood cell count, renal function, culture results, echocardiographic findings, hemodynamics, Acute Physiology and Chronic Health Evaluation (APACHE) II score, and the need for mechanical ventilation including non-invasive positive pressure ventilation.

### Determination of the final diagnosis

The frequency of RHC examinations for estimating the PCWP has now decreased, because there is no clear evidence of benefit [8,9]. We therefore determined the final diagnosis according to the clinical features and responses to treatments. As shown in Table 1, the clinical diagnostic criteria were originally defined to ensure the objectivity of determining the final diagnosis.

**Table 1:** Criteria for the clinical diagnosis

No.	Clinical features	CPE	ALI/ARDS
1	PCWP in right heart catheterization (if examined), mmHg	> 18	≤ 18
2	Culture results and/or immunological test for infections	Negative	Positive
3	LVEF < 50% and/or diastolic dysfunction on echocardiography	Present	Absent
4	Pleural effusion on chest radiographs	Present	Absent
5	PaO_2 _/ FiO_2 _> 400 within 3 days after diuresis	Present	Absent

CPE subjects were required No. 1 and No.2, together with at least two features in Nos. 3-5.

ALI/ARDS subjects were required No.1, together with at least three features in Nos. 2-5.

The patients who did not meet these diagnostic criteria were categorized as having mixed edema,

ALI: acute lung injury; ARDS: acute respiratory distress syndrome; CPE: cardiogenic pulmonary edema; LVEF: left ventricular ejection fraction; PCWP: pulmonary capillary wedge pressure

First of all, if RHC was examined, the measured value was required for each diagnosis, CPE or ALI/ARDS [2]. Next, the evidence for infection, culture result and/or immunological test was required. This requirement must be cautiously considered. The subjects with ALI/ARDS include non-infectious causes such as pancreatitis or trauma [2], moreover, not all infections are always confirmed by culture results and/or immunological tests. As a result, this condition was set as a requirement for the diagnosis of ALI/ARDS. In contrast, for the diagnosis of CPE in the present study, this condition was set as an absolute prerequisite in order to rule out the patients with CPE which was triggered by infections as much as possible. Because, CPE triggered by infections could correspond to mixed-type edema. Immunological tests for infections included polymerase chain reaction assays for *Pneumocystis jiroveci *and acid-fast bacillus, a Cytomegalovirus antigenemia assay, and rapid detection kits for serum *Mycoplasma pneumoniae *antibody and urinary antigens of *Streptococcus pneumoniae *and *Legionella pneumophila*. In additon, heart failure was divided to two types, namely systolic dysfunction or diastolic dysfunction [34], and therefore the condition of No. 3 (Table 1) was stipulated. The presence of pleural effusion on chest radiographs, which was more frequently seen in patients with CPE than those with ALI/ARDS [13,14], and an improvement in the respiratory status with diuresis [15] were also included in these criteria. However, each finding was not absolute for determining the diagnosis. We therefore established multiple options, the CPE subjects were required No. 1 and No. 2, together with at least two features in Nos. 3-5, ALI/ARDS ones were required No.1, together with at least three features in Nos. 2-5. The patients who did not meet these diagnostic criteria were categorized as mixed edema, and thus were excluded from the analyses in this study. These classifications were done by two independent physicians who were blinded to the BNP and CRP data. Of the 147 total enrolled cases, 124 met the above-mentioned criteria, the other 23 cases did not meet the criteria. As a result, 71 patients were diagnosed with CPE, 53 patients with ALI/ARDS, and 23 patients with mixed type pulmonary edema.

### Measurements of BNP and CRP

BNP levels in plasma were measured immediately after the sample collection in the emergency room with a well-validated commercially available immunoassay (Tosoh, Tokyo, Japan) with a detection limit of 4 pg/mL. CRP levels in plasma were measured by a standard sensitive Latex-immunoassay (Denka Seiken, Tokyo, Japan) with a detection limit of 0.1 mg/L. The normal range for this assay is < 10 mg/L. Both values in all patients were measured within 2 hrs after arriving at the emergency department.

### Statistical analysis

Statistical analyses were performed using the PASW statistics 18.0 software package (IBM SPSS, Tokyo, Japan), except for comparison of the receiver operating characteristic (ROC) curve, which was performed using the STATA version 11 software package (Stata, College Station, TX, US). Statistical significance was defined by a p value < 0.05 for all analyses. Continuous variables were tested for normality using the Shapiro-Wilk test, and compared using the Student's *t-*test or Mann-Whitney test distribution. The chi-square test was applied for comparing categorical variables, unless one of the categories had fewer than 20 observations, in which case, the Fisher's exact test was applied. For statistically different findings between CPE and ALI/ARDS groups, we used multiple logistic regression analysis to compare the relevant outcomes. Continuous variables were redefined as dichotomous variables using the medians as cut-off values in the study population, excluding mixed edema. The sensitivity, specificity, positive predictive value, negative predictive value, and diagnostic accuracy rates were calculated according to standard definitions. The accuracy of discriminatory performance was compared by the area under ROC curves. The mixed type patients were excluded for all statistical analyses, such as multiple logistic regression and ROC curves with determination of the cut-off levels of BNP and CRP.

## Results

### Patient characteristics

Baseline characteristics and the results of laboratory and clinical examinations of the 147 patients, who were stratified according to their final diagnosis, are shown in Table 2 and 3. The ALI/ARDS subjects included 7 patients with ALI and 46 patients with ARDS. Between the ALI/ARDS and CPE groups, there was no statistical difference in the age, gender, and frequency of a past history of cardiac or pulmonary diseases. In the evaluation of patients who had affectors for CRP producibility, there were three patients (5.7%) in ALI/ARDS, and seven patients (9.9%) in CPE, who had acute hepatic failure. There were five patients (9.4%) in ALI/ARDS, and two patients (2.8%) in CPE, who received corticosteroids. No significant differences were observed between these prevalence rates (p = 0.31, p = 0.12, respectively). The patients with CPE were more likely to present with high systolic and diastolic blood pressure. All patients with ALI/ARDS had high APACHE II scores, in line with the findings of previous reports [16,22]. Echocardiography was performed in 96% of patients, but RHC was performed in only 13 cases (9%). The etiology of the 53 patients with ALI/ARDS consisted of 30 with intrapulmonary diseases [including 20 cases of acute pneumonia (38%) and 10 of aspiration pneumonia (19%)] and 23 patients with extrapulmonary diseases [including 20 with sepsis (38%), 1 with burns, 1 with severe pancreatitis, and 1 due to trauma].

**Table 2:** Baseline patient characteristics at the time of enrollment*

	ALI/ARDS (*n *= 53)	CPE (*n *= 71)	Mixed (*n *= 23)	p values for ALI/ARDS vs CPE
Patient demographics				
age, yrs	78 (69-85)	81 (74-89)	81 (75-89)	0.083
male gender	34 (64)	35 (49)	13 (57)	0.100
History of cardiac disease				
chronic heart failure	18 (34)	36 (51)	16 (70)	0.063
myocardial infarction	9 (17)	18 (25)	5 (22)	0.264
angina pectoris	4 (8)	7 (10)	2 (9)	0.654
prior PCI	3 (6)	7 (10)	3 (13)	0.606
prior CABG	4 (8)	5 (7)	3 (13)	0.915
History of diabetes mellitus	9 (17)	21 (30)	7 (30)	0.105
History of pulmonary disease				
COPD	5 (9)	2 (3)	0 (0)	0.114
asthma	1 (2)	1 (1)	1 (4)	0.834
Physical examination				
systolic blood pressure, mmHg	120 (100-146)	148 (126-170)	126 (110-156)	< 0.001
diastolic blood pressure, mmHg	67 (54-83)	86 (70-100)	80 (64-89)	< 0.001
heart rate, beats/min	100 (79-113)	108 (90-119)	104 (90-136)	0.109
body temperature,°F	98.6 (97.0-99.9)	98.1 (97.0-98.8)	98.6 (97.5-100.8)	0.483
S3 gallop	2 (4)	11 (15)	5 (22)	0.031

*Data are expressed as the number (%) or median (interquartile range).

ALI: acute lung injury; ARDS: acute respiratory distress syndrome; CABG: coronary artery bypass graft; COPD: chronic obstructive pulmonary disease; CPE: cardiogenic pulmonary edema; PCI: percutaneous coronary intervention; PCWP: pulmonary capillary wedge pressure

**Table 3:** Findings of laboratory and clinical examinations*

	ALI/ARDS (*n *= 53)	CPE (*n *= 71)	Mixed (*n *= 23)	p value for ALI/ARDS vs CPE
Laboratory findings				
WBC count, ×1000/μL	10.4 (7.3-12.8)	9.6 (7.2-13.4)	9.8 (5.9-11.2)	0.587
CRP, mg/L	119 (62-165)	8 (2-42)	100 (36-184)	< 0.001
BNP, pg/mL	202 (95-439)	691 (416-1194)	403 (221-1048)	< 0.001
GFR, mL/min	60.8 (28.6-92.9)	46.0 (35.9-58.4)	56.2 (29.7-86.9)	0.056
PaO_2_/FiO2	100 (68-147)	111 (82-156)	166 (9-220)	0.322
Mechanical ventilation	34 (64)	35 (49)	9 (39)	0.100
APACHE II score	17 (16-19)	17 (16-18)	17 (16-18)	0.245
Positive culture results	28 (53)	0 (0)	0 (0)	--
Chest radiography				
number of patients examined	53 (100)	71 (100)	23 (100)	--
infiltrates on two quadrants	24 (45)	38 (54)	18 (78)	0.468
infiltrates on four quadrants	28 (53)	33 (46)	6 (26)	0.604
pleural effusion	43 (81)	66 (93)	19 (83)	0.043
cardiomegaly	25 (47)	39 (55)	13 (57)	0.500
Echocardiography				
number of patients examined	50 (94)	71 (100)	20 (87)	--
LVEF, %	60 (56-66)	57 (45-69)	49 (39-65)	0.052
mitral regurgitation ≥grade 2	15/50 (30)	31/71 (44)	7/23 (30)	0.182
right ventricular dilation	13/50 (26)	29/71 (41)	8 (35)	0.135
Hemodynamics				
number of patients examined	6 (11)	4 (6)	3 (10)	0.206
PCWP, mmHg	10 (9-13)	32 (28-37)	32 (26-34)	0.096

*Data are expressed as the number (%) or median (interquartile range).

ALI: acute lung injury; APACHE: acute physiology and chronic health evaluation; ARDS: acute respiratory distress syndrome; BNP: brain natriuretic peptide; CRP: C-reactive protein; CPE: cardiogenic pulmonary edema; GFR: glomerular filtration rate; LVEF: left ventricular ejection fraction; PCWP: pulmonary capillary wedge pressure

### BNP in patients with pulmonary edema

**Figure 1:**
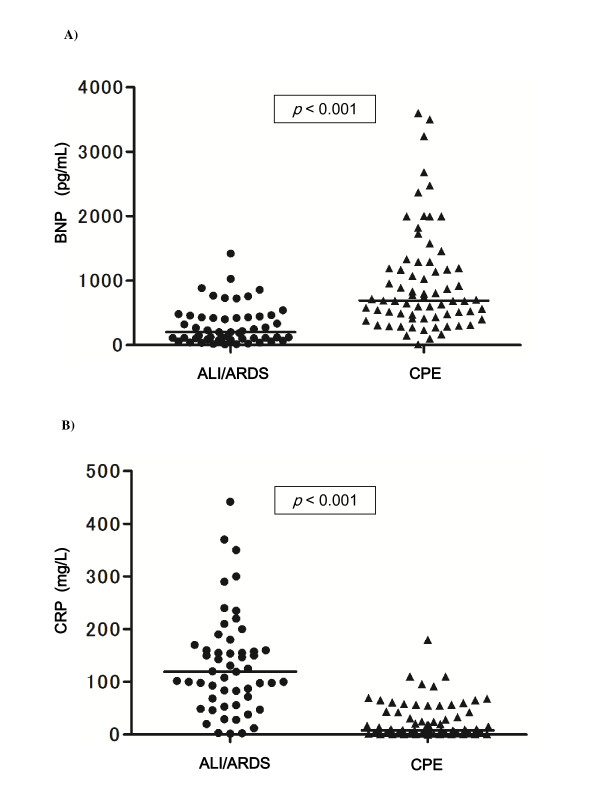
**Plasma concentrations of brain natriuretic peptide (BNP) A) and C-reactive protein (CRP) B) in patients with cardiogenic pulmonary edema (CPE) (*n *= 71), or acute lung injury/acute respiratory distress syndrome (ALI/ARDS) with (*n *= 53)**. The p values show between these subjects. The BNP levels in CPE patients were higher than those in ALI/ARDS patients (*p *< 0.001). The CRP levels in the ALI/ARDS patients were higher than those in the CPE patients (*p *< 0.001).

**Figure 2:**
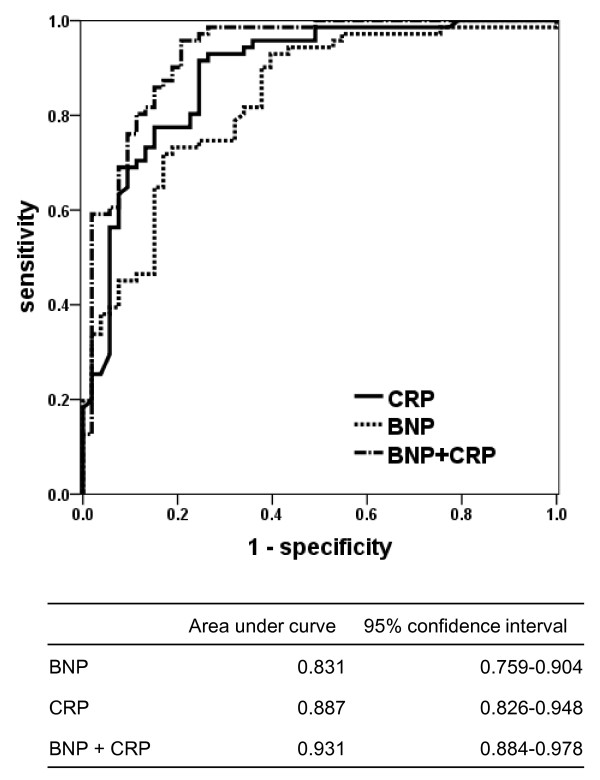
**Receiver operating characteristics curve (AUC) analyses of brain natriuretic peptide (BNP), C-reactive protein (CRP), and BNP combined with CRP in discriminating cardiogenic pulmonary edema (CPE) from acute lung injury/acute respiratory distress syndrome (ALI/ARDS), excluding the mixed type of pulmonary edema**. CRP levels were converted to the negative values, because lower CRP levels were expected to be more common in CPE patients. *p *< 0.001 compared BNP alone with combination BNP and CRP; *p *= 0.030 compared CRP alone with BNP and CRP.

As shown in Table 3 and Figure 1A, the initial levels of plasma BNP were significantly different between the patients with CPE and ALI/ARDS. When patients with ALI/ARDS were subclassified into those with sepsis or without sepsis, no significant differences were observed between the median (interquartile range; IQR) BNP levels in patients with sepsis [299 (128-463) pg/mL] and those without sepsis [115 (70-417) pg/mL]. The area under the ROC curve (Figure 2) when BNP was used to differentiate CPE from ALI/ARDS was 0.831 (p < 0.001). A BNP cutoff value of 500 pg/mL (approximate values as the highest likelihood ratio according to the ROC curves, excluding the mixed type edema) had a sensitivity of 69.0%, a specificity of 83.1%, and an accuracy of 75.0% for detecting CPE (Table 4).

**Table 4:** Performance characteristics of various cut off points of BNP or CRP, excluding mixed type edema

Cut-off points	Sensitivity	Specificity	PPV	NPV	Accuracy
BNP levels for CPE					
≧400	80.3	66.0	76.0	71.4	74.2
≧500	69.0	83.1	84.5	66.7	75.0
≧600	60.6	84.9	84.3	61.6	71.0

CRP levels for ALI/ARDS					
≧80	53.5	90.6	88.4	59.3	69.4
≧50	59.2	69.8	72.4	56.1	63.7
≧20	69.0	50.9	65.3	55.1	61.3

Cut-off points of BNP (pg/mL), CRP (mg/L), (%)

ALI: acute lung injury; ARDS: acute respiratory distress syndrome; BNP: brain natriuretic peptide; CRP: C-reactive protein; CPE: cardiogenic pulmonary edema; NPV: negative predictive value; PPV: positive predictive value

### CRP in patients with pulmonary edema

The initial levels of plasma CRP in patients with ALI/ARDS were significantly higher than those with CPE (Table 3 and Figure 1 B). The area under the ROC curve (Figure 2) when CRP was used to differentiate CPE from ALI/ARDS was 0.887 (p < 0.001). A CRP cutoff value of 50 mg/L (approximate values as the highest likelihood ratio according to the ROC curves, excluding the mixed type edema) had a sensitivity of 59.2%, a specificity of 69.8%, and an accuracy of 63.7% for detecting ALI/ARDS (Table 4).

### Predictors of the diagnosis after adjusting for other variables

By means of multiple logistic-regression analyses, we determined the additional diagnostic power of measurement of BNP and CRP, patients' age, systolic blood pressure, S3 gallop, left ventricular ejection fraction, and the presence of pleural effusion on the chest radiograph. In order to increase the statistical power, continuous variables were redefined as dichotomous variables using the following cut-off values: age of 80 years, systolic blood pressure of 135 mmHg, left ventricular ejection fraction of 60% (median of population, respectively), and plasma levels of CRP 50 mg/L and BNP of 500 pg/mL (approximate values as the highest likelihood ratio according to the ROC curves, excluding the mixed type edema). This model showed that higher levels of BNP and lower levels of CRP were strong independent predictors of CPE (Table 5).

**Table 5:** Predictors for distinguishing CPE from ALI/ARDS, excluding the mixed type*

		univariate			multivariate	
	
Predictors for CPE	OR	95%CI	p-value	OR	95%CI	p-value
age > 80y	1.350	0.660-2.761	0.411	1.125	0.370-3.417	0.836
systolic BP > 135 mmHg	3.148	1.496-6.627	0.003	3.999	1.311-12.198	0.015
S3 gallop sound	4.032	0.845-19.251	0.080	9.142	0.727-115.032	0.087
CRP > 50 mg/L	0.083	0.035-0.196	< 0.001	0.106	0.035-0.323	< 0.001
BNP > 500 pg/mL	12.50	5.057-30.898	< 0.001	14.425	4.382-47.483	< 0.001
LVEF > 60%	0.474	0.223-1.006	0.052	0.799	0.258-2.476	0.697
Pleural effusion on chest radiograph	2.805	0.881-8.932	0.081	5.293	0.791-35.434	0.086

*Results of multiple logistic regression analysis.

ALI: acute lung injury; ARDS: acute respiratory distress syndrome; BNP: brain natriuretic peptide; BP: blood pressure; CRP: C-reactive protein; CPE: cardiogenic pulmonary edema; LVEF: left ventricular ejection fraction

### The value of combination measurements of BNP and CRP in patients with pulmonary edema

As shown in Figure 2, the area under the ROC curve when the combination of BNP and CRP was used to differentiate CPE from ALI/ARDS was 0.931 (p < 0.001). There was no significant difference in the area under the ROC curve between BNP alone and CRP alone (p = 0.201), while the combination of BNP and CRP offered excellent performance compared with BNP alone (p < 0.001) and CRP alone (p = 0.030). There was a moderate correlation between the levels of BNP and CRP (ρ = -0.414).

## Discussion

This study is the first to demonstrate that a combination of the measurement of BNP and CRP levels provides an advantage over measurement of BNP or CRP levels alone for the differential diagnosis of CPE and ALI/ARDS.

The present study demonstrated that BNP had good diagnostic utility for distinguishing CPE from ALI/ARDS, consistent with the previous reports [15,22]. The AUC of BNP measurement for the diagnosis in our study appeared to be higher than those in these previous studies. This might have been due to the timing of the examination. The BNP levels of all patients in our study were measured within 2 hrs after visiting the emergency room, while the median time from the recognition of pulmonary edema to the measurement of BNP was 3 hrs (IQR; 0.5 to 14) in the report by Rana et al [15]. BNP levels generally decrease after treatment for heart failure [35], hence, the high levels and accuracy of our study may be explained by our measurement of BNP levels in most patients before starting treatment.

Several authors also have reported that BNP levels cannot discriminate CPE from sepsis-induced ARDS [23-25], because the plasma BNP level may increase due to myocardial dysfunction or the direct effect of inflammatory mediators produced by myocytes in patients with sepsis, in spite of their normal cardiac function [36]. Our study showed no significant differences in the plasma BNP levels between cases of ALI/ARDS with sepsis and those without sepsis. However, the present study population was relatively small, so it may be difficult to discriminate CPE and ALI/ARDS by using BNP alone if the rate of sepsis is high. Additionally, the BNP levels are also known to be elevated in part as a result of the acute right heart dysfunction that is associated with ARDS [37,38]. Increased stretching of the right ventricle and atrium may cause BNP release, independent of left ventricular filling pressure, in patients with ARDS. In the present study, the frequency of right ventricular dilation/hypokinesis when evaluated by the right heart load in echocardiography was not significantly different between CPE and ALI/ARDS patients. However, the evaluation using echocardiography was clinically difficult, and this was one limitation associated with this study. If right heart dysfunction caused by ALI/ARDS influences the plasma BNP levels, a differential diagnosis of CPE versus ALI/ARDS would be extremely difficult using the BNP level alone.

We also demonstrated the usefulness of measuring CRP for distinguishing CPE from ALI/ARDS. Some patients with ALI/ARDS could have severe community acquired pneumonia (CAP) cases with a score of 4 or 5 based on the CURB65 severity score for CAP [39]. Recent studies demonstrated that CRP is an independent marker of the severity of CAP [40,41]. Therefore, the CRP levels in patients with ALI/ARDS, including severe pneumonia, may be useful for distinguishing these cases from patients with CPE. Although the individual measurements of BNP or CRP are effective for differentiating ALI/ARDS from CPE, we found the combination measurement of BNP and CRP to provide better results compared with measuring either BNP or CRP alone. Because the BNP level can increase in patients with sepsis, our results suggest that measuring both CRP and BNP can eliminate this drawback to the measurement of BNP alone. Therefore, this combination measurement will help physicians determine a differential diagnosis for critically ill patients with pulmonary edema, even if the patients are suspected to have sepsis or acute cor pulmonale induced by ALI/ARDS. As the value of RHC, echocardiography, and the measurement of BNP alone for the differential diagnosis is still controversial, our results suggest that a combination of the measurements of BNP and CRP may therefore be an effective additional or alternative, non-invasive, and inexpensive diagnostic strategy.

This study has several limitations. First, this study validated clinical diagnoses, because an objective "gold standard" method for diagnosis of ALI/ARDS is absent. Although we performed a multiple logistic regression analysis and showed that BNP was an independent predictor, the possibility of collinearity between each surrogate feature (such as PCWP or LVEF as listed in the clinical diagnostic criteria) and BNP cannot be completely ruled out. Second, there was a relatively high number of mixed-type cases of pulmonary edema, and these cases were excluded for the statistical analyses. Finally, this study was limited to a still small sample size at a single center.

In clinical practice, we occasionally provide treatment concurrently targeting both CPE and ALI/ARDS for critical patients. This is important in several cases, however, we must continue to challenge the differential diagnosis of pulmonary edema in order to provide an optimal treatment. Better diagnoses will lead to better treatment and thereby contribute to better patient outcomes.

## Conclusions

This is the first report evaluating the utility of measuring both CRP and BNP in plasma to provide a differential diagnosis in patients with pulmonary edema. Our results indicate that measurement of CRP could be useful as well as BNP for discriminating ALI/ARDS from CPE. In addition, the evaluation of the combination of CRP and BNP can provide an even higher accuracy for the diagnosis. It is hoped that a large multi-center survey including cases of sepsis-induced ARDS can be accomplished in the near future.

## List of abbreviations

ALI: acute lung injury; APACHE: acute physiology and chronic health evaluation; ARDS: acute respiratory distress syndrome; AUC: area under the curve; BNP: brain natriuretic peptide; CI: confidence interval; COPD: chronic obstructive pulmonary disease; CPE: cardiogenic pulmonary edema; LVEF: left ventricular ejection fraction; IQR: interquartile range; OR: odds ratio; PCWP: pulmonary capillary wedge pressure; RHC: right heart catheterization; ROC: receiver operating characteristic.

## Competing interests

All of the authors explicitly declare that there are no conflicts of interest in connection with this article.

## Authors' contributions

KK, HI and ST designed this study and drafted the manuscript. OT and NE participated in the design of the study and performed the statistical analysis. OY, NE, JM, YH and JK conceived of the study, and participated in its design and coordination and helped to draft the manuscript. All authors read and approved the final manuscript.

## References

[B1] Brower RG Ware LB Berthiaume Y Matthay MA Treatment of ARDS Chest 2001 120 1347 1367 10.1378/chest.120.4.1347 11591581

[B2] Bernard GR Artigas A Brigham KL Carlet J Falke K Hudson L Lamy M Legall JR Morris A Spragg R The American-European Consensus Conference on ARDS. Definitions, mechanisms, relevant outcomes, and clinical trial coordination Am J Respir Crit Care Med 1994 149 818 824 7509706 10.1164/ajrccm.149.3.7509706

[B3] Sloane PJ Gee MH Gottlieb JE Albertine KH Peters SP Burns JR Machiedo G Fish JE A multicenter registry of patients with acute respiratory distress syndrome. Physiology and outcome Am Rev Respir Dis 1992 146 419 426 1489134 10.1164/ajrccm/146.2.419

[B4] Monnet X Anguel N Osman D Hamzaoui O Richard C Teboul JL Assessing pulmonary permeability by transpulmonary thermodilution allows differentiation of hydrostatic pulmonary edema from ALI/ARDS Intensive Care Med 2007 33 448 453 10.1007/s00134-006-0498-6 17221189

[B5] Fein A Grossman RF Jones JG Overland E Pitts L Murray JF Staub NC The value of edema fluid protein measurement in patients with pulmonary edema Am J Med 1979 67 32 38 10.1016/0002-9343(79)90066-4 463915

[B6] Eisenberg PR Jaffe AS Schuster DP Clinical evaluation compared to pulmonary artery catheterization in the hemodynamic assessment of critically ill patients Crit Care Med 1984 12 549 553 10.1097/00003246-198407000-00001 6734221

[B7] Mermel LA Maki DG Infectious complications of Swan-Ganz pulmonary artery catheters. Pathogenesis, epidemiology, prevention, and management Am J Respir Crit Care Med 1994 149 1020 1036 8143037 10.1164/ajrccm.149.4.8143037

[B8] Sandham JD Hull RD Brant RF Knox L Pineo GF Doig CJ Laporta DP Viner S Passerini L Devitt H Kirby A Jacka M Canadian Critical Care Clinical Trials Group A randomized, controlled trial of the use of pulmonary-artery catheters in high-risk surgical patients N Engl J Med 2003 348 5 14 10.1056/NEJMoa021108 12510037

[B9] Harvey S Harrison DA Singer M Ashcroft J Jones CM Elbourne D Brampton W Williams D Young D Rowan K collaboration PA-Ms Assessment of the clinical effectiveness of pulmonary artery catheters in management of patients in intensive care (PAC-Man): a randomised controlled trial Lancet 2005 366 472 477 10.1016/S0140-6736(05)67061-4 16084255

[B10] Connors AF Jr Speroff T Dawson NV Thomas C Harrell FE Jr Wagner D Desbiens N Goldman L Wu AW Califf RM Fulkerson WJ Vidaillet H Jr Broste S Bellamy P Lynn J Knaus WA The effectiveness of right heart catheterization in the initial care of critically ill patients. SUPPORT Investigators JAMA 1996 276 889 897 10.1001/jama.276.11.889 8782638

[B11] Montgomery AB Stager MA Carrico CJ Hudson LD Causes of mortality in patients with the adult respiratory distress syndrome Am Rev Respir Dis 1985 132 485 489 4037521 10.1164/arrd.1985.132.3.485

[B12] Zimmerman GA Morris AH Cengiz M Cardiovascular alterations in the adult respiratory distress syndrome Am J Med 1982 73 25 34 10.1016/0002-9343(82)90920-2 7091171

[B13] Milne EN Pistolesi M Miniati M Giuntini C The radiologic distinction of cardiogenic and noncardiogenic edema AJR Am J Roentgenol 1985 144 879 894 3872571 10.2214/ajr.144.5.879

[B14] Aberle DR Wiener-Kronish JP Webb WR Matthay MA Hydrostatic versus increased permeability pulmonary edema: diagnosis based on radiographic criteria in critically ill patients Radiology 1988 168 73 79 3380985 10.1148/radiology.168.1.3380985

[B15] Rana R Vlahakis NE Daniels CE Jaffe AS Klee GG Hubmayr RD Gajic O B-type natriuretic peptide in the assessment of acute lung injury and cardiogenic pulmonary edema Crit Care Med 2006 34 1941 1946 10.1097/01.CCM.0000220492.15645.47 16691132

[B16] Levitt JE Vinayak AG Gehlbach BK Pohlman A Van Cleve W Hall JB Kress JP Diagnostic utility of B-type natriuretic peptide in critically ill patients with pulmonary edema: a prospective cohort study Crit Care 2008 12 R3 10.1186/cc6764 18194554 PMC2374600

[B17] Ware LB Matthay MA Clinical practice. Acute pulmonary edema N Engl J Med 2005 353 2788 2796 10.1056/NEJMcp052699 16382065

[B18] Nagueh SF Kopelen HA Zoghbi WA Feasibility and accuracy of Doppler echocardiographic estimation of pulmonary artery occlusive pressure in the intensive care unit Am J Cardiol 1995 75 1256 1262 10.1016/S0002-9149(99)80772-3 7778550

[B19] Mueller C Scholer A Laule-Kilian K Martina B Schindler C Buser P Pfisterer M Perruchoud AP Use of B-type natriuretic peptide in the evaluation and management of acute dyspnea N Engl J Med 2004 350 647 654 10.1056/NEJMoa031681 14960741

[B20] Davis M Espiner E Richards G Billings J Town I Neill A Drennan C Richards M Turner J Yandle T Plasma brain natriuretic peptide in assessment of acute dyspnoea Lancet 1994 343 440 444 10.1016/S0140-6736(94)92690-5 7905953

[B21] Maisel AS Krishnaswamy P Nowak RM McCord J Hollander JE Duc P Omland T Storrow AB Abraham WT Wu AH Clopton P Steg PG Westheim A Knudsen CW Perez A Kazanegra R Herrmann HC McCullough PA Breathing Not Properly Multinational Study, Investigators: Rapid measurement of B-type natriuretic peptide in the emergency diagnosis of heart failure N Engl J Med 2002 347 161 167 10.1056/NEJMoa020233 12124404

[B22] Karmpaliotis D Kirtane AJ Ruisi CP Polonsky T Malhotra A Talmor D Kosmidou I Jarolim P de Lemos JA Sabatine MS Gibson CM Morrow D Diagnostic and prognostic utility of brain natriuretic Peptide in subjects admitted to the ICU with hypoxic respiratory failure due to noncardiogenic and cardiogenic pulmonary edema Chest 2007 131 964 971 10.1378/chest.06-1247 17426196 PMC2278171

[B23] Januzzi JL Morss A Tung R Pino R Fifer MA Thompson BT Lee-Lewandrowski E Natriuretic peptide testing for the evaluation of critically ill patients with shock in the intensive care unit: a prospective cohort study Crit Care 2006 10 R37 10.1186/cc4839 16507171 PMC1550815

[B24] Rudiger A Gasser S Fischler M Hornemann T von Eckardstein A Maggiorini M Comparable increase of B-type natriuretic peptide and amino-terminal pro-B-type natriuretic peptide levels in patients with severe sepsis, septic shock, and acute heart failure Crit Care Med 2006 34 2140 2144 10.1097/01.CCM.0000229144.97624.90 16763507

[B25] Tung RH Garcia C Morss AM Pino RM Fifer MA Thompson BT Lewandrowski K Lee-Lewandrowski E Januzzi JL Utility of B-type natriuretic peptide for the evaluation of intensive care unit shock Crit Care Med 2004 32 1643 1647 10.1097/01.CCM.0000133694.28370.7F 15286538

[B26] Okamura JM Miyagi JM Terada K Hokama Y Potential clinical applications of C-reactive protein J Clin Lab Anal 1990 4 231 235 10.1002/jcla.1860040316 2112596

[B27] Lobo SM Lobo FR Bota DP Lopes-Ferreira F Soliman HM Melot C Vincent JL C-reactive protein levels correlate with mortality and organ failure in critically ill patients Chest 2003 123 2043 2049 10.1378/chest.123.6.2043 12796187

[B28] Smith RP Lipworth BJ Cree IA Spiers EM Winter JH C-reactive protein. A clinical marker in community-acquired pneumonia Chest 1995 108 1288 1291 10.1378/chest.108.5.1288 7587431

[B29] Ware LB Pathophysiology of acute lung injury and the acute respiratory distress syndrome Semin Respir Crit Care Med 2006 27 337 349 10.1055/s-2006-948288 16909368

[B30] Bajwa EK Khan UA Januzzi JL Gong MN Thompson BT Christiani DC Plasma C-reactive protein levels are associated with improved outcome in ARDS Chest 2009 136 471 480 10.1378/chest.08-2413 19411291 PMC2818414

[B31] Global Initiative for Asthma Global strategy for asthma management and prevention 2008 http://www.ginasthma.com Accessed December 2, 2008

[B32] Global Initiative for Chronic Obstructive Lung Disease Global Strategy for the Diagnosis, Management, and Prevention of Chronic Obstructive Pulmonary Disease 2010 http://www.goldcopd.com/GuidelinesResources.asp?l1=2&l2=0 Accessed April 23, 2010

[B33] Hanley JA McNeil BJ The meaning and use of the area under a receiver operating characteristic (ROC) curve Radiology 1982 143 29 36 7063747 10.1148/radiology.143.1.7063747

[B34] Grossman W Diastolic dysfunction in congestive heart failure N Engl J Med 1991 325 1557 1564 10.1056/NEJM199111283252206 1834939

[B35] Gackowski A Isnard R Golmard JL Pousset F Carayon A Montalescot G Hulot JS Thomas D Piwowarska W Komajda M Comparison of echocardiography and plasma B-type natriuretic peptide for monitoring the response to treatment in acute heart failure Eur Heart J 2004 25 1788 1796 10.1016/j.ehj.2004.07.038 15474693

[B36] Charpentier J Luyt CE Fulla Y Vinsonneau C Cariou A Grabar S Dhainaut JF Mira JP Chiche JD Brain natriuretic peptide: A marker of myocardial dysfunction and prognosis during severe sepsis Crit Care Med 2004 32 660 665 10.1097/01.CCM.0000114827.93410.D8 15090944

[B37] Vieillard-Baron A Page B Augarde R Prin S Qanadli S Beauchet A Dubourg O Jardin F Acute cor pulmonale in massive pulmonary embolism: incidence, echocardiographic pattern, clinical implications and recovery rate Intensive Care Med 2001 27 1481 1486 10.1007/s001340101032 11685341

[B38] Nagaya N Nishikimi T Okano Y Uematsu M Satoh T Kyotani S Kuribayashi S Hamada S Kakishita M Nakanishi N Takamiya M Kunieda T Matsuo H Kangawa K Plasma brain natriuretic peptide levels increase in proportion to the extent of right ventricular dysfunction in pulmonary hypertension J Am Coll Cardiol 1998 31 202 208 9426041 10.1016/s0735-1097(97)00452-x

[B39] Lim WS van der Eerden MM Laing R Boersma WG Karalus N Town GI Lewis SA Macfarlane JT Defining community acquired pneumonia severity on presentation to hospital: an international derivation and validation study Thorax 2003 58 377 382 10.1136/thorax.58.5.377 12728155 PMC1746657

[B40] Chalmers JD Singanayagam A Hill AT C-reactive protein is an independent predictor of severity in community-acquired pneumonia Am J Med 2008 121 219 225 10.1016/j.amjmed.2007.10.033 18328306

[B41] Almirall J Bolibar I Toran P Pera G Boquet X Balanzo X Sauca G Community-Acquired Pneumonia Maresme Study G Contribution of C-reactive protein to the diagnosis and assessment of severity of community-acquired pneumonia Chest 2004 125 1335 1342 10.1378/chest.125.4.1335 15078743

